# From image to insight: an AI-enabled framework for echocardiography acquisition, reconstruction, interpretation and interaction

**DOI:** 10.1093/ehjdh/ztaf141

**Published:** 2025-12-04

**Authors:** Ziqiang Zhou, Qingmiao Yang

**Affiliations:** Cardiovascular Center, Beijing Tongren Hospital, Capital Medical University, Dongjiaominxiang 1#, Dongcheng District, Beijing 100730, China; Cardiovascular Center, Beijing Tongren Hospital, Capital Medical University, Dongjiaominxiang 1#, Dongcheng District, Beijing 100730, China

**Keywords:** Echocardiography, Artificial intelligence, Acquisition guidance, Generative models and digital twins, Image-free/imageless diagnostics, Human–AI interaction

## Abstract

This narrative, perspective-style review proposes a structured framework for how artificial intelligence (AI) may reshape key steps of the echocardiography workflow. We argue that AI’s main contribution is not only to automate existing tasks but to enable new approaches to data acquisition, reconstruction, interpretation, and human–system interaction. We first summarize clinically integrated and, where available, regulated AI solutions for echocardiography, including acquisition guidance, view recognition, and automated chamber/function quantification. We then outline four AI-enabled directions that are at varying stages of maturity: (i) reconstruction, in which generative models could derive more complete, four-dimensional cardiac representations from sparse ultrasound data; (ii) acquisition, where AI may serve as a real-time co-pilot to optimize information content rather than image aesthetics; (iii) interpretation, extending to ‘image-free’ models that learn directly from upstream radiofrequency/channel data; and (iv) interaction, using semantic or augmented-reality interfaces to reduce clinician cognitive load and improve situated decision-making. Together, these developments point to a gradual shift from subjective, image-centric reading towards more quantitative, data-driven echocardiography. Their realization will depend on prospective validation, fit-for-purpose regulatory pathways, and safeguards for fairness and safety, especially for generative and image-free paradigms. Our goal is to map these possibilities and to distinguish evidence-supported applications from those that remain conceptual.

## Graphical Abstract

**Graphical Abstract:**
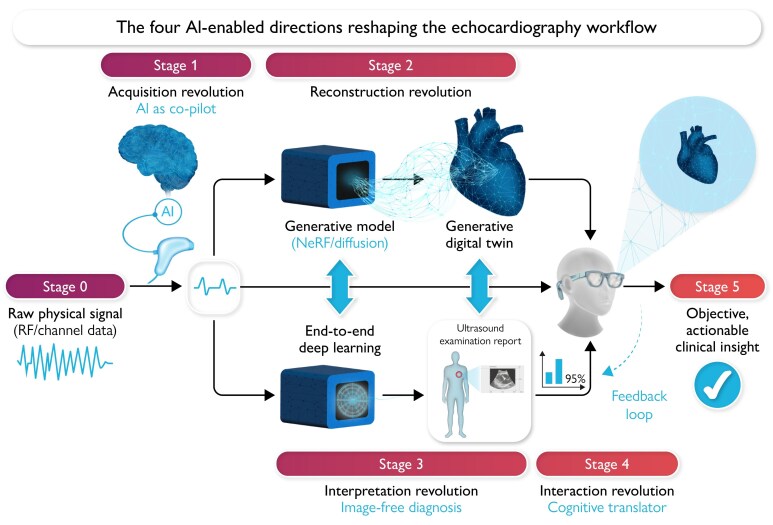

## Introduction: from artisan to analyst, and beyond the enduring paradox of echocardiography

Echocardiography is the undisputed cornerstone of cardiovascular diagnostics, prized for its safety, accessibility, real-time nature, and cost-effectiveness.^1^ Yet, its profound clinical utility is fundamentally constrained by an enduring paradox: it is a modality rich in quantitative physical information that is ultimately filtered through the narrow, subjective bandwidth of human perception and motor skill. For decades, the field has grappled with the consequences of this artisan-dependent nature, including significant operator dependency, high inter- and intra-observer variability in key measurements, and the inherently subjective quality of image interpretation.^2^ These limitations, formally acknowledged in professional guidelines, can compromise diagnostic certainty and impact patient management, representing a ceiling on the modality’s potential.^3–5^

### The first wave of AI: critiquing the mimicry paradigm

In response to these challenges, the first wave of artificial intelligence, powered by deep learning, has made remarkable and necessary inroads. AI models now offer real-time acquisition guidance to novice users,^6^ automate view classification with expert-level accuracy,^7^ and provide fully automated quantification of left ventricular ejection fraction (LVEF) and strain, with prospective randomized trials demonstrating non-inferiority to human experts and significant workflow efficiencies.^8,9^ These achievements are invaluable, representing a concerted effort to standardize outputs and reduce the well-documented variability that plagues manual analysis.^10,11^

However, this initial wave, while clinically useful, represents a ‘dimensional reduction’ of AI’s true potential. It has largely focused on tasking a powerful, non-human intelligence with mimicking the outputs of a flawed, human-centric workflow. It seeks to optimize the existing paradigm but fails to question its fundamental assumptions. The vast majority of AI research to date has focused on solving the *consequences* of echocardiography's core limitations (e.g. correcting measurement variability) rather than addressing the *source* of those limitations—the subjective, manual processes of data acquisition and visual interpretation.

### The central thesis: AI as a paradigm critic and thought catalyst

This review posits that the next, and far more transformative, era of AI in echocardiography will be defined by a shift in its role: from mimicking human tasks to enabling new, AI-supported conceptual approaches. The true value of AI lies not in its ability to replicate human tasks more quickly or consistently, but in its capacity to process data at scales and in dimensions inaccessible to human cognition, thereby prompting researchers to revisit our most foundational assumptions and ask first-principle questions: Why are we fundamentally limited to interpreting a grayscale B-mode image? Why do we optimize acquisition parameters for the subjective appeal to the human eye? Why do we reconstruct a volume from slices instead of generating a complete, dynamic whole from sparse data? By embracing AI as a collaborative partner, we may unlock its potential to catalyse new scientific directions *within echocardiography*.

### Roadmap: four AI-enabled directions

**Figure 1:**
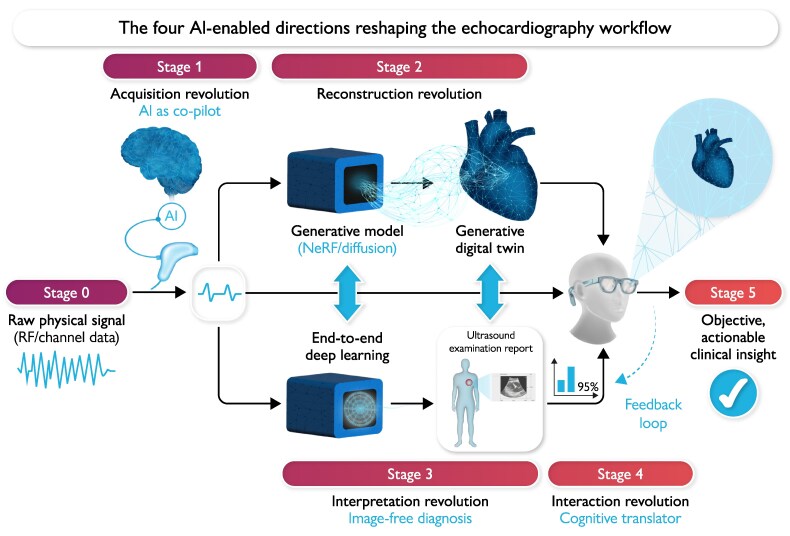
AI-enabled echocardiography workflow. Raw physical signal (Stage 0) is acquired and optimized by AI as a real-time co-pilot (Stage 1). The optimized data then feed two parallel pathways: generative reconstruction to create a digital cardiac representation (Stage 2), and end-to-end deep learning for image-free diagnosis (Stage 3). Outputs are fused and presented through human–AI interaction tools (Stage 4) to deliver objective, actionable clinical insight (Stage 5). Several of these applications operate upstream of the conventional B-mode image.

For clarity, we organize this perspective into four AI-enabled ‘revolutions’—a term we use descriptively to indicate shifts in where AI acts in the echocardiography workflow, not to imply that these changes are inevitable or already realized. Each direction addresses a core limitation of the traditional paradigm by offering an AI-enabled alternative (*Table 1*). We discuss, in turn, reconstruction, acquisition, interpretation, and interaction. Together, these directions outline a pathway from qualitative, image-centric echocardiography towards more quantitative and accessible practice (*Figure 1*).

**Table 1:** Four AI-enabled directions in echocardiography and their AI catalysts

The revolution	The old paradigm (The problem)	The new paradigm (The AI-catalysed solution)	Key AI enablers	Ultimate clinical transformation
**Reconstruction**	Sparse 2D slices; challenging 3D stitching with artifacts and low temporal resolution.	Generative 4D models creating a complete, dynamic heart from sparse, incomplete data.	Neural radiance fields (NeRFs), diffusion models, GANs.	Patient-specific cardiac digital twins for *in silico* trials and personalized procedural planning.
**Acquisition**	Manual, subjective parameter tuning (gain, focus, etc.) optimized for human visual perception.	Real-time, intelligent parameter optimization driven by a defined reward signal to maximize information density.	Reinforcement learning (RL), control theory.	Maximally informative data capture at the source, reducing downstream errors, and enhancing diagnostic robustness.
**Interpretation**	Subjective human visual assessment of heavily processed, lossy B-mode images.	‘Imageless diagnostics’ where AI learns directly from raw, pre-beamformed acoustic data (RF/channel data).	End-to-end deep learning, signal processing networks.	Objective, quantitative diagnosis based on underlying physics, potentially uncovering novel, non-visual biomarkers.
**Interaction**	High cognitive load; abstract grayscale data on a separate screen (‘split-attention effect’).	Real-time semantic visualization, rendering complex data as intuitive overlays and animations.	Real-time segmentation, augmented reality (AR).	Drastically reduced cognitive load, democratized expertise, and a new language for human–AI collaboration.

### Literature search and selection strategy (non-PRISMA)

This is a narrative, thesis-driven review and therefore does not follow a systematic PRISMA protocol. Our aim was to map emerging AI-enabled directions in echocardiography rather than to exhaustively synthesize all available evidence. We conducted a non-systematic search of PubMed/MEDLINE, IEEE Xplore, and arXiv for publications from January 2009 to March 2025 using combinations of the following terms: ‘echocardiography’, ‘ultrasound’, ‘artificial intelligence’, ‘deep learning’, ‘acquisition guidance’, ‘generative AI’, ‘diffusion model’, ‘NeRF’, ‘digital twin’, ‘reinforcement learning’, and ‘large language model’. Reference lists of key cardiology/imaging society guidelines were hand-searched to identify additional studies. We prioritized studies that (i) reported methodological milestones in AI for echocardiography, (ii) provided prospective or multi-centre validation, (iii) described regulated or commercially available solutions, or (iv) introduced concepts directly relevant to our four-part framework. Regulatory and implementation sources that are still evolving were included when they were the only available references, and are labelled as such in the text.

## The revolution in reconstruction: from the ‘ultrasound CT’ dream to the generative reality of cardiac digital twins

### Critique of the current paradigm

For decades, echocardiography has presented clinicians with a series of two-dimensional (2D), tomographic ‘keyhole’ views of a complex, three-dimensional (3D), and rapidly moving organ. While experts are trained to mentally integrate these disparate slices into a coherent anatomical model, the process is inherently incomplete and subjective. Conventional 3D echocardiography, though an improvement, has been hindered by significant challenges, including stitching artefacts from multiple cardiac cycles, low temporal and spatial resolution, and persistent operator dependence, which have limited its routine clinical adoption.^3^ The long-held dream of an ‘ultrasound computed tomography (CT)’—a modality that could capture the entire heart in a single, comprehensive volume—has remained elusive due to these technical and practical barriers.

### The generative leap: neural radiance fields (NeRFs)

Generative AI offers a path to leapfrog these limitations entirely. The first key technology is the neural radiance field (NeRF), a method that reconceptualizes 3D representation.^12^ Instead of building a discrete model (like a voxel grid or mesh), a NeRF learns a continuous, five-dimensional neural representation of a scene, mapping any 3D spatial co-ordinate (*x,y,z*) and 2D viewing direction (*θ, ϕ*) to a colour and density value.^12^ Trained on a collection of 2D images with known camera poses, a NeRF can synthesize novel, photorealistic views from any arbitrary viewpoint through a process of volumetric rendering.^13^

When applied to ultrasound, this technology holds the potential to take a series of tracked 2D sweeps and generate a complete, continuous 3D volume that can be sliced and viewed from any angle, far surpassing the quality of traditional stitching algorithms.^14^ Pioneering work such as Ultra-NeRF has begun to adapt this framework specifically for ultrasound by incorporating a physics-informed rendering model that accounts for view-dependent phenomena unique to acoustic imaging, such as anisotropic reflections and attenuation.^15^ However, significant challenges remain, particularly in adapting NeRFs to a dynamic, deforming object like the heart, which requires robust methods for handling cardiac and respiratory motion and precise probe tracking.^16,17^

### The generative leap: diffusion models and GANs for shape synthesis

A complementary class of generative models, including diffusion models and generative adversarial networks (GANs), offers a powerful approach not just for rendering views but for synthesizing entire 3D anatomical shapes.^18^ These models learn the underlying statistical distribution of a training dataset and can then generate novel, realistic samples from that learned distribution.

Recent work has demonstrated the power of latent diffusion models (LDMs) for generating high-fidelity 3D cardiac meshes. Architectures like MeshLDM can be trained on datasets of patient-specific heart models to produce diverse and anatomically plausible new instances, capturing the complex geometric variations seen across populations and disease states.^18^ This capability is crucial for augmenting limited datasets, especially for rare diseases, thereby improving the robustness of other machine learning models.^19^ Similarly, GANs can be employed for sophisticated domain translation tasks, such as generating a fully labelled 3D echocardiographic volume from a high-resolution anatomical model derived from CT, effectively creating perfectly annotated synthetic data for training segmentation algorithms.^20^

### The new clinical reality: the cardiac digital twin

The convergence of these generative technologies enables the creation of a true, patient-specific, four-dimensional cardiac digital twin—a dynamic, computational replica of an individual’s heart.^21^ This is not merely a static 3D model but a queryable, interactive ‘holographic sandbox’ that can be manipulated and analysed in ways previously confined to science fiction. The clinical implications are transformative:

- **Personalized procedural planning:** Surgeons and interventionalists can perform virtual ‘fly-throughs’ of a patient’s unique anatomy, meticulously planning valve replacements or complex congenital heart repairs.
- **Predictive device simulation:** The fit and function of devices like transcatheter valves, closure devices, or clips can be simulated *in silico*, predicting potential complications like paravalvular leak or device embolization before the patient enters the catheterization laboratory.
- ***In silico* clinical trials:** By generating populations of ‘digital siblings”—anatomical variants based on a patient's digital twin—researchers can test new drugs or devices on a virtual cohort, exploring the impact of subtle anatomical variations on outcomes and dramatically accelerating the pace of medical innovation.^22,23^

This shift from deterministic reconstruction to probabilistic generation fundamentally alters the nature of the 3D model. The model is no longer a direct interpolation *of* the acquired data but a plausible, learned instantiation *conditioned on* that data. This allows the model to intelligently ‘fill in’ information that was never captured, which is the source of its power. However, it also introduces the risk of ‘hallucinated’ features, a key concern for clinical adoption.^1^ This redefines the very concept of diagnostic fidelity. Fidelity is no longer about how perfectly the model represents the acquired pixels, but about the biological and anatomical plausibility of the *generated* components. This creates a profound new challenge for validation. A robust framework for evaluating synthetic echocardiography data is therefore essential, assessing not only image-level fidelity (realism) but also downstream utility (e.g. does segmentation or quantification perform similarly on synthetic vs. real data?) and fairness (does the model generate diverse anatomies without amplifying biases present in the training set?).

## The revolution in acquisition: from manual artistry to AI as a ‘co-pilot’ for real-time optimization

### Critique of the current paradigm

The current echocardiography workflow positions AI as a passive, downstream analyst. The quality of the data it receives is entirely dependent on the manual artistry of a human operator, who adjusts a complex array of machine parameters—gain, focus, frequency, and compression—to achieve an ‘image quality’ standard that is defined by subjective, anthropocentric criteria.^2^ This process is inherently variable and suboptimal. Even the most advanced AI guidance systems, which excel at helping operators achieve standard probe *positions*, do not actively optimize the intrinsic *parameters* of the ultrasound beam itself to maximize the underlying information content.^1^ AI is left to analyse data that were optimized for a different, less capable interpreter: the human eye.

### The front-end shift: AI as an active ‘co-pilot’

This section proposes a radical paradigm shift: moving AI from the back end (analysis) to the front end (acquisition). We envision an AI that functions as an active ‘co-pilot’, engaging in a real-time collaborative loop with the human sonographer. In this model, the human performs the high-level tasks of patient interaction and gross probe positioning, while the AI undertakes the complex, high-frequency, multi-dimensional task of real-time parameter optimization.

### Technical elaboration: a reinforcement learning (RL) framework

This human–AI collaborative control problem is ideally suited to a reinforcement learning (RL) framework, a class of machine learning that excels at learning optimal strategies for sequential decision-making through trial and error.^24^ The system can be conceptualized as follows:

- **Agent:** The AI control model.
- **Environment:** The patient’s acoustic window, the dynamic cardiac anatomy, and the physics of ultrasound propagation.
- **Actions:** The agent can execute a vast array of actions at microsecond speed, such as dynamically adjusting transmit frequency, focal depth, sector width, gain, dynamic range, and even advanced beamforming strategies.
- **State:** The agent’s perception of the environment is the incoming raw ultrasound data stream (e.g. RF data).
- **Reward:** This is the most critical and revolutionary component. The reward signal is not a measure of aesthetic image quality. Instead, it is a quantitative measure of ‘information density’ or ‘diagnostic utility’. For example, the reward could be defined as maximizing the confidence score of a downstream diagnostic algorithm (e.g. an amyloidosis detector), maximizing the clarity of the endocardial border to improve segmentation accuracy, or maximizing the signal-to-noise ratio of a specific tissue property being measured.

By optimizing for a task-specific reward, the AI learns a control policy that ensures the acquired data are maximally informative for the clinical question at hand.^25^ This approach draws on the success of RL in other complex, real-time control domains like robotic surgery and autonomous navigation, where agents learn robust policies in dynamic environments.^26,27^

### The new clinical reality: redefining image quality

This paradigm shift forces a redefinition of ‘image quality’. An optimal image is no longer one that appears clear, well-contrasted, and low-noise to the human eye. Instead, an optimal acquisition is one that contains the maximal amount of machine-readable information required to answer a clinical question with the highest possible confidence. This could lead to acquisitions that appear unconventional or even noisy to a human observer but are information-rich for an AI model. The clinical transformation is a human–AI team that acquires a diagnostically optimal dataset at the source, every time, dramatically reducing the influence of poor image quality on diagnostic accuracy and improving the robustness of all subsequent analyses.^2^ This evolution from a human-operated tool to a robotic sensing platform, where the AI has deep, granular control over the physics of signal generation, will likely drive a co-evolution in ultrasound hardware, demanding more software-defined, AI-addressable controls on future machines.

## The revolution in interpretation: beyond visual constraints towards ‘imageless diagnostics’

### Critique of the current paradigm

The most significant, yet unexamined, limitation in medical imaging AI may be the image itself. The B-mode echocardiogram is a heavily processed, log-compressed, envelope-detected representation of a far more complex physical reality. It is an artefact of history, optimized for the limited dynamic range and feature-detection capabilities of the human visual system. In the conversion from the raw acoustic signal to the greyscale image, a vast amount of quantitative physical information—particularly phase, frequency, and full dynamic range—is irretrievably discarded.^28^ Forcing AI to interpret this lossy, human-centric data format is akin to asking a supercomputer to analyse a musical masterpiece by looking only at a blurry photograph of the sheet music.

### The disruptive leap to raw data

The third revolution, therefore, involves bypassing the B-mode image entirely. The future of AI-driven interpretation lies in developing end-to-end models that learn directly from the raw, pre-beamformed channel data or the beamformed radiofrequency (RF) data.^29,30^ These raw data contain the complete, complex-valued signal from each transducer element, preserving the full amplitude and phase information of the returning acoustic waves.^31^ This is a data domain of immense richness and dimensionality that is completely inaccessible to human interpretation but is perfectly suited for deep learning.

### Technical elaboration: learning from physics

Processing these types of data is not a standard computer vision task. It requires novel deep learning architectures designed to handle complex-valued, high-frequency signals and to understand the underlying physics of wave propagation, scattering, and tissue interaction. Early studies have already demonstrated the profound potential of this approach. In ultrasound elastography, for instance, deep learning models that estimate tissue strain directly from RF signals have been shown to achieve higher signal-to-noise and contrast-to-noise ratios than state-of-the-art methods that operate on B-mode images.^32,33^ In this paradigm, the AI model is not learning to recognize anatomical shapes in a picture; it is learning to recognize the complex, high-dimensional spatio-temporal signatures of physiological and pathological states as they are encoded directly in the acoustic physics.

### The new clinical reality: ‘imageless diagnostics’

This approach culminates in the concept of ‘imageless diagnostics’. The AI’s output is not an image, a segmentation, or a measurement for a human to interpret. Instead, the output is a direct, quantitative diagnostic or prognostic prediction (e.g. ‘probability of cardiac amyloidosis: 95%’, ‘predicted 1-year mortality: 25%’). This could finally solve the core problem of subjectivity that has plagued ultrasound since its inception. The diagnosis would be based on objective, physical data, not a qualitative interpretation of a greyscale image.

Furthermore, this method has the potential to uncover entirely novel disease biomarkers. An AI model might discover subtle, distributed signatures within the RF data—changes in backscatter statistics, phase coherence, or spectral content—that correspond to early-stage disease processes like myocardial infiltration in amyloidosis or interstitial fibrosis in heart failure with preserved ejection fraction, long before any structural changes become visible on a standard B-mode image. This aligns with the demonstrated ability of some commercial AI tools to detect hidden disease patterns from standard images, but takes the concept a step further by tapping into a much richer data source.^34^ This paradigm, however, fundamentally breaks the traditional validation loop. If there is no image for a human expert to review, we can no longer validate the AI based on concordance.

Validation must shift entirely to correlation with hard clinical outcomes, demanding a new evidentiary framework. This requires rigorous, prospective, multi-center randomized controlled trials with pre-registered protocols and clearly defined primary clinical endpoints (e.g. all-cause mortality, heart failure hospitalizations, or major adverse cardiac events). The performance of an ‘imageless’ algorithm should not be judged on accuracy alone, but on its clinical utility, which can be assessed using methods like decision curve analysis to determine if it leads to better clinical decision-making than existing standards. Establishing the robustness of such a paradigm will be a monumental task, but it is the necessary path to achieving truly objective diagnostics.

## The revolution in interaction: from ‘white noise’ to the cognitive leap of real-time semantic visualization

### Critique of the current paradigm

Interpreting a standard echocardiogram is a cognitively demanding task, even for seasoned experts. It requires the mental integration of multiple 2D greyscale views into a dynamic 3D model, tracking complex motion, and inferring physiological function from subtle visual cues. For a non-expert, the screen often appears as indecipherable ‘white noise’. This high cognitive load is a significant barrier to training, proficiency, and the modality’s broader application in point-of-care settings. A key contributor to this cognitive burden is the ‘split-attention effect’, where an operator must constantly shift their focus and mental mapping between the patient’s body, the position of the probe in their hand, and an abstract data representation on a separate monitor.^35,36^

### AI as the ‘cognitive translator’

The fourth revolution positions AI as the crucial bridge between the complex physics of ultrasound and the intuitive, powerful pattern-recognition system of the human brain. Here, AI’s role is not to replace the human interpreter, but to serve as a real-time ‘cognitive translator’, rendering abstract, hard-to-decipher data into a format that is immediately and intuitively understandable. The goal is to reduce extraneous cognitive load, allowing the clinician to dedicate their mental resources to higher-level reasoning and decision-making.^37^

### Technical elaboration: semantic segmentation and augmented reality (AR)

Two key technologies enable this revolution:

- **Real-time semantic segmentation:** Lightweight, efficient deep learning models, such as specialized U-Nets, can now run in real-time on modern ultrasound platforms. These models can automatically identify and delineate key anatomical structures on a frame-by-frame basis—for example, tracing the endocardial border, highlighting valve leaflets, or identifying papillary muscles.^9^ This segmentation can be augmented with functional information, such as overlaying a colour map representing real-time myocardial strain or wall thickening velocity.
- **Augmented reality (AR):** AR devices, such as head-mounted displays, can take this semantic information and overlay it directly onto the operator’s field of view.^38^ This directly mitigates the split-attention effect by co-locating the ultrasound data with the physical patient and the operator’s hands.^35^ Instead of looking at a separate screen, the clinician sees the segmented, colour-coded heart beating directly within the patient’s chest.

#### The new clinical reality: reducing cognitive load and democratizing expertise

The clinical impact of this approach is two-fold. First, for the expert, it dramatically reduces cognitive load. Instead of mentally tracking the endocardium to assess for wall motion abnormalities, they can instantly perceive regions of hypokinesis as a persistent blue on a colour-coded strain map, freeing up cognitive bandwidth for more complex clinical integration.^35^ Second, it helps to democratize expertise. A complex interventional procedure, such as guiding a catheter to a specific cardiac location, is made significantly easier and safer when the AI provides an AR overlay showing the target anatomy, the trajectory of the device, and nearby critical structures to avoid.^39^ This could substantially shorten the learning curve for both basic echocardiography and advanced procedures, improving the standardization of skill and expanding the capabilities of a wider range of clinicians. This real-time visualization also creates a powerful, implicit feedback loop for human–AI collaboration. By seeing the AI’s interpretation (e.g. its segmentation) overlaid on the live image, the human operator can immediately spot discrepancies and correct them, creating a synergistic partnership that is far more powerful than a simple post-hoc analysis.

## Discussion: navigating the path from revolution to clinical reality

The convergence of these four revolutions promises to fundamentally reshape echocardiography. However, translating this visionary future into a safe and effective clinical reality requires navigating a complex landscape of practical, regulatory, and ethical challenges. This requires moving beyond simplistic notions of AI implementation to embrace more sophisticated frameworks for validation, oversight, and governance as summarized in *Table 2*. These approaches are still being operationalized by regulators and professional societies, and should be regarded as illustrative rather than definitive pathways, especially for generative or adaptive AI systems.

**Table 2:** Illustrative frameworks for responsible clinical implementation of advanced AI (pathways under active development)

Domain	Key challenge	State-of-the-art approach/Principle	Relevance to the four revolutions
**Validation strategies**	From retrospective concordance to prospective clinical utility.	Outcome-based randomized controlled trials (RCTs); real-world evidence generation.^8^	Essential for validating the clinical impact of ‘imageless diagnostics’ and AI-driven acquisition.
**Regulatory pathways**	Regulating adaptive and generative models that evolve over time.	FDA’s TPLC approach; predetermined change control plans; robust post-market surveillance.^40,41^	Critical for the safe deployment of RL-based ‘co-pilots’ and ensuring the fidelity of generative digital twins.
**Human–AI collaboration**	Moving beyond ‘black box’ veto to synergistic partnership.	Integrated HITL architectures with XAI, uncertainty quantification, and feedback mechanisms.^42–44^	Enables clinical trust in AR-based semantic interaction and provides necessary oversight for all four revolutions.
**Ethical safeguards**	Mitigating dataset bias and preventing the amplification of health disparities.	Proactive fairness audits; diverse and representative data sourcing; bias mitigation^45^ algorithms.	Prevents generative models from creating biased digital twins and ensures diagnostic models perform equitably.

AI, artificial intelligence; AR, augmented reality; FDA, U.S. Food and Drug Administration; HITL, human-in-the-Loop; RCTs, randomized controlled trials; RL, reinforcement learning; TPLC, total product life cycle; XAI, explainable AI.

### The human–AI symbiosis: beyond simple oversight

The traditional ‘human-in-the-loop’ model, which often implies a clinician simply vetoing a black-box AI recommendation, is insufficient for the paradigms we propose. A more deeply integrated model of **human–AI collaboration** is required, built on three pillars^43^:

- **Explainability (XAI):** To build trust, AI systems must provide insight into their reasoning. This must move beyond simple heatmaps to more interactive forms of explanation, such as the real-time semantic visualizations discussed previously, which allow a clinician to see the world through the AI’s ‘eyes’.^42,44^
- **Uncertainty quantification:** A safe AI is one that knows what it does not know. Systems must be designed to output not just a prediction, but also a calibrated confidence score. This enables intelligent task allocation, automatically flagging low-confidence or out-of-distribution cases for mandatory human expert review, thereby optimizing workflow efficiency without compromising safety.^44^
- **Continuous feedback integration:** Clinical workflows must be designed to capture expert feedback in a structured way, allowing for continuous and safe refinement of model performance over time, ideally within a robust regulatory framework.^44^

### Validation, regulation, and trust

The revolutionary nature of these new AI paradigms demands an evolution in our approach to validation and regulation. This was a key concern.^1^

- **Regulatory landscape:** Regulatory bodies like the U.S. Food and Drug Administration (FDA) are actively developing frameworks for AI/ML-based Software as a Medical Device.^40^ The FDA’s proposed Total Product Life Cycle approach, which includes concepts like a Predetermined Change Control Plan, is a crucial step towards regulating adaptive algorithms that learn over time, though it stops short of allowing true continuous, autonomous learning in the clinical environment.^41^
- **Generative AI risks:** Generative models introduce unique and serious risks that regulators are beginning to address. These include the potential for models to ‘hallucinate’ clinically meaningful but factually incorrect information, the challenge of ensuring diagnostic fidelity in synthetic data, and the risk of misuse.^46,47^
- **Commercial pathfinders:** The path to clinical integration is being paved by pioneering companies that have successfully navigated this complex regulatory environment.^1^ These commercial successes provide a real-world roadmap for translating advanced AI from code to clinic (*Table 3*).
- **The imperative for prospective validation:** Ultimately, retrospective studies demonstrating concordance with human experts are insufficient. The true clinical utility of these tools—especially disruptive ones like imageless diagnostics—can only be established through rigorous, prospective, randomized clinical trials that measure their impact on hard clinical outcomes, patient management, and healthcare costs. The trial by He *et al*. on AI-assisted LVEF assessment serves as a landmark example of the high level of evidence required.^8^

**Table 3:** Regulated and commercially available AI solutions in echocardiography

System/Company	Key function(s)	Regulatory pathway (US FDA)	Key evidence/Note
**Caption Guidance**/Caption Health	Real-time AI guidance for image acquisition	De Novo (DEN190040), 2020^48^	Demonstrated ability to enable non-specialists to acquire diagnostic-quality images.
**Us2.v1**/Us2.ai	Automated quantification; detection of aortic stenosis & cardiac amyloidosis	510(k) (K210791), 2021; (K223020), 2023^49,50^	Provides a fully automated workflow from view selection to reporting.
**EchoGo**/Ultromics	Detection of HFpEF & cardiac amyloidosis; strain analysis	510(k) (K213463), 2022; (K222134), 2022^34^	Leverages AI to identify subtle disease patterns often missed by visual assessment.

AI, artificial intelligence; FDA, U.S. Food and Drug Administration; HFpEF, heart failure with preserved ejection fraction. De Novo and 510(k) are regulatory pathways for medical devices overseen by the FDA.

### Ethical imperatives: bias and fairness

A critical prerequisite for the responsible deployment of any clinical AI is a proactive approach to mitigating algorithmic bias. Models trained on data from limited demographic or geographic populations can underperform on underrepresented groups, perpetuating and even amplifying existing health disparities.^45^ Ensuring fairness requires a commitment throughout the AI lifecycle to using diverse and representative training datasets, conducting regular fairness audits to detect and correct performance disparities across subgroups, and developing new techniques for bias mitigation.

## Conclusion: future directions and clinical implementation

Artificial intelligence in echocardiography is moving beyond the automation of routine tasks towards enabling analyses that are difficult for humans to perform alone. AI has the potential to complement, rather than replace, human perception and cognition in this setting.

The four AI-enabled directions outlined in this review—reconstruction, acquisition, interpretation, and interaction—indicate a gradual shift from subjective, image-centric workflows towards more quantitative, data-driven, and ultimately more accessible echocardiography. A plausible future configuration is an AI co-pilot that optimizes acquisition at the source, routes information-rich raw ultrasound data to image-free diagnostic models, and links these outputs to a patient-specific, visualizable digital representation delivered through intuitive human–AI interfaces. This scenario is conceptual and will require stepwise technical, clinical, and regulatory validation.

Realizing such an evolution will depend on prospective studies, fit-for-purpose regulatory pathways, and robust human-in-the-loop governance, especially for generative and image-free paradigms. Framed this way, AI is not a replacement for expert echocardiographers but a means to make echocardiography more precise, more objective, and more widely usable.

Box 1 The interface with multimodal foundation models and LLMs

While our four revolutions focus on the ultrasound data domain, they will inevitably interface with the broader ecosystem of multimodal foundation models and large language models (LLMs) (Box 1). These models, which unify text, images, and structured data into a single representational space, will primarily connect with the interpretation and interaction revolutions. For example, vision-language models like EchoCLIP have shown zero-shot capabilities in interpreting echocardiograms, suggesting a future where a single generalist model could perform multiple diagnostic tasks. The most immediate applications will be in automated report generation and natural language interfaces for querying study findings. In the longer term, these models could couple with the ‘imageless diagnostics’ engine to provide text-based explanations for its predictions or interact with the ‘digital twin’ to simulate and describe the outcomes of hypothetical interventions, creating a truly conversational diagnostic partner.

## Data Availability

No new data were generated or analysed in support of this research.
